# Performance of bioelectrical impedance analysis compared to dual X-ray absorptiometry (DXA) in Veterans with COPD

**DOI:** 10.1038/s41598-022-05887-4

**Published:** 2022-02-04

**Authors:** Paola N. Cruz Rivera, Rebekah L. Goldstein, Madeline Polak, Antonio A. Lazzari, Marilyn L. Moy, Emily S. Wan

**Affiliations:** 1 Pulmonary, Allergy, Sleep and Critical Care Medicine Section, VA Boston Healthcare System, 1400 VFW Parkway, West Roxbury, Boston, MA 02132 USA; 2 Primary Care and Rheumatology Sections, VA Boston Healthcare System, Boston, MA USA; 3 Harvard Medical School, Boston, MA USA; 4 Boston University School of Medicine, Boston, MA USA; 5 Channing Division of Network Medicine, Brigham and Women’s Hospital, Boston, MA USA

**Keywords:** Diagnostic markers, Preclinical research

## Abstract

We examined the performance of a commercially-available handheld bioimpedance (BIA) device relative to dual X-ray absorptiometry (DXA) to assess body composition differences among Veterans with chronic obstructive pulmonary disease (COPD). Body composition was measured using DXA and BIA (Omron HBF-306C) at a single time point. Correlations between BIA- and DXA-assessed percent fat, fat mass, and fat-free mass were analyzed using Spearman (ρ) and Lin Concordance Correlation Coefficients (ρ_c_). Mean differences in fat mass were visualized using Bland–Altman plots. Subgroup analyses by obesity status (BMI < 30 versus ≥ 30) were performed. Among 50 participants (96% male; mean age: 69.5 ± 6.0 years), BIA-assessed fat mass was strongly correlated (ρ = 0.94) and demonstrate excellent concordance (ρ_c_ = 0.95, [95%CI: 0.93–0.98]) with DXA, with a mean difference of 2.7 ± 3.2 kg between BIA and DXA. Although Spearman correlations between BIA- and DXA-assessed percent fat and fat-free mass were strong (ρ = 0.8 and 0.91, respectively), concordance values were only moderate (ρ_c_ = 0.67 and 0.74, respectively). Significantly stronger correlations were observed for obese relative to non-obese subjects for total percent fat (ρ_obese_ = 0.85 versus ρ_non-obese_ = 0.5) and fat mass (ρ_obese_ = 0.96 versus ρ_non-obese_ = 0.84). A handheld BIA device demonstrated high concordance with DXA for fat mass and moderate concordance for total percent fat and fat-free mass.

ClinicalTrials.gov: NCT02099799.

## Introduction

Chronic obstructive pulmonary disease (COPD), a leading cause of morbidity and mortality worldwide^1^, is characterized by airways obstruction, dyspnea, and reduced exercise capacity. Unintentional weight loss and low body mass index (BMI) have been independently associated with increased mortality risk in COPD patients^2,3^. Importantly, differences in body composition, such as disproportionate loss of muscle mass, are associated with worse exercise capacity and health-related quality of life in patients with COPD^4,5^. Early and accurate detection of differences in body composition may facilitate timely institution of appropriate management, such as improved nutrition and pulmonary rehabilitation.

Body composition measurements can be ascertained using a number of methods. The dual X-ray absorptiometry (DXA) scan is currently the gold standard measure of body composition; however, DXA scan is costly, may only be available in limited settings, is time-consuming, and involves exposure to ionizing radiation. Other methods such as skinfold thickness, hydro-densitometry, and bioimpedance analysis (BIA) have been utilized as alternatives to the DXA scan to clinically measure body composition^6,7^. BIA devices offer rapid, non-invasive assessments of percent total body fat based on the determination of total body water content. Given the ease of obtaining BIA measurements relative to DXA, the majority of studies of body composition in COPD patients have been conducted using BIA. However, variability in the performance of different BIA devices in measuring body composition in patients with COPD exists, with limited data available on the accuracy of portable and handheld devices^7–9^.

In the current work, we compared the accuracy of body composition measurements, including percent fat, fat mass, and fat-free mass, between the Omron HBF-306C (Omron Healthcare Inc., Bannockburn, IL, USA), a commercially available handheld BIA device that emits an extremely low electrical current of 50 kHz and 500 µA, relative to the DXA scan. The Omron HBF-306C BIA device provides measurement of percent total body fat, from which fat mass and fat-free mass (FFM) can be derived from concurrent anthropomorphic measurements (height, weight). For additional comparison, we determined FFM using anthropomorphic measurements alone (age, sex, height, weight) based on validated protocols^10,11^ and compared the performance to DXA-assessed FFM.

In addition to our primary comparisons between BIA- and DXA-assessed measures of body composition, we performed several pre-specified secondary and subgroup analyses. First, because electrode placement can impact BIA measurements^8,12^, we examined the correlation between percent regional body fat measures (i.e., trunk, gynoid, android, arms, and legs.) obtained from the DXA scan and BIA device mean percent total body fat. Second, because the performance of BIA devices may vary at the extremes of BMI^6^, we compared the correlations (1) between BMI and DXA relative to BMI and BIA and (2) between BIA- and DXA-assessed body composition by obesity status.

## Methods

We analyzed data from a subset of participants enrolled in a physical activity intervention study (ClinicalTrials.gov: NCT02099799)^13^ recruited from VA Boston Healthcare System. The protocol (#2791) was approved by the VA Boston Healthcare System Committee on Human Research Institutional Review Board; all studies were performed in accordance with the relevant guidelines and regulations and written informed consent was obtained from all participants. All subjects had COPD, defined as the ratio of forced expiratory volume in the first second and forced vital capacity (FEV_1_/FVC)  < 0.70 on clinical spirometry or chest computed tomography (CT) scan evidence of emphysema by radiologist report, and were recruited from the outpatient pulmonary clinics from 2015 to 2019. A DXA scan and BIA assessments were completed on the same day at either the baseline or 12-month visit; measurements were performed only once in each subject, thus, these data were considered cross-sectional. Exclusion criteria for the intervention study included: inability to ambulate with or without assistance, hypoxemia (defined as oxygen saturation < 85%) during the 6-min walk test, and participation in a pulmonary rehabilitation program at the time of screening. In addition, participants with a pacemaker or other implanted electronic device were excluded from BIA assessments.

A 5th generation GE Healthcare iDXA dual x-ray absorptiometry (DXA) scanner (GE Lunar, Madison, WI, USA) was used to perform total body composition and regional analysis of fat according to manufacturer’s standard procedures in the supine position with the scan mode automatically determined by the device. Subjects were wearing a hospital gown or basic underwear and had all metal objects removed. All DXA scans were performed by the same manufacturer-certified operator following the operator’s manual for patient positioning and data acquisition. Body composition analysis was performed using enCORE Software version 13.60.033, according to manufacturer’s standard protocol. The regions of interest (ROIs) for the regional fat analysis were automatically determined by the enCORE software. The trunk region was defined by cuts passing through the shoulder joints, a line below the shin and as close to the body as possible, with the lower boundary defined by cuts through the femoral necks. The lower boundary of the android region, was defined by a horizontal cut through the pelvis at the level of the iliac crests. The upper boundary of the android region was defined upwards to 20% of the distance between the pelvis and shoulders and laterally to the border of the body. The gynoid region upper boundary was defined at the iliac crests with the lower boundary extending below the pelvis downward 1.5 times the height of the android region to the upper leg cuts^12^. All imaging was subsequently interpreted and analyzed by a Certified Clinical Densitometrist (AAL) who verified and when necessary repositioned the ROIs based on the cut line instructions provided in the enCORE operator’s manual. The iDXA unit was evaluated daily using the GE Lunar block, supplied by the manufacturer, for quality assurance which ensured that the device was operating within manufacturer’s specifications.

BIA measurements of percent body fat were made in the seated position and were repeated in triplicate using two different BIA devices of the same model (Omron HBF-306C). Unless noted otherwise, the mean from all six BIA measurements were used in the analyses. Participants were asked to void immediately prior to the BIA assessment since the impedance value is a function of the resistance of electrical current against water flow.

Reproducibility between (1) repeated measurements using the same device and (2) average measurements between two different BIA devices was assessed using the intra- and inter-device coefficients of variation. BMI was calculated using weight and height derived from DXA measurements; obesity was defined as a BMI ≥ 30. Anthropomorphic measurements included measuring weight and height using a digital or balance scale and a stadiometer, respectively. BIA-derived fat mass (in kilograms) was calculated as [measured weight, kg] x [BIA % body fat/100]. BIA-derived FFM was calculated as [measured weight, kg] – [BIA fat mass, kg]. Anthropomorphic FFM was calculated using age, sex, measured height, and measured weight to determine total body water (TBW) content using the equations of Watson et al.^11^; anthropomorphic FFM then calculated as TBW/0.73^10^. The co-primary outcomes for comparison between BIA and DXA were (1) total percent body fat, (2) fat mass (in kg), and (3) fat-free mass (in kg). Correlations between BIA- and DXA-assessed body composition and anthropomorphic FFM were examined using Spearman correlations and the Lin Concordance Correlation Coefficient (CCC)^14^ with DXA as the gold standard. The difference in percent fat and fat mass measurements obtained from DXA and BIA was visualized using Bland–Altman plots^15^. Secondary analyses examining the correlation between BIA-assessed total percent body fat and DXA-assessed regional body fat percentage were assessed using Spearman correlations. We examined the performance of BIA relative to DXA separately by obesity status (BMI < 30 and ≥ 30) using Spearman correlations; significance testing to compare correlations was performed using the z-scores. Interactions with obesity status were assessed using a multiplicative term in multivariable-adjusted regression models. A p-value < 0.05 was considered significant.

## Results

**Figure 1:**
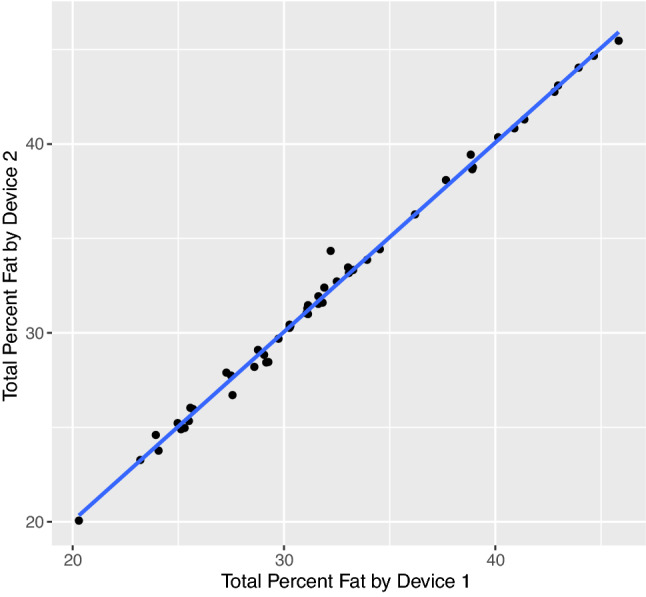
Correlation between mean percent body fat measurements obtained using two bioimpedance devices. Scatter plot with best fit line (solid blue) of percent body fat measurements taken using two different Omron HBF-306C bioimpedance devices. Measurements were taken in triplicate using each advice. Mean of three measurements from each device is shown. Spearman correlation between devices was 0.996 (p < 0.0001).

Baseline characteristics of the subjects (n = 50) included in the analysis are shown in Table 1. The cohort was predominantly male and of White race with moderate airflow obstruction. Percent total body fat measurements from the two BIA devices were highly reproducible, with intra-device coefficients of variation (CoV) ranging from 0.8 to 0.9% and an inter-device CoV of 0.1%. Consistent with these findings, the Spearman correlation between devices was also very strong (ρ = 0.996, p < 0.0001, Fig. 1).

**Table 1:** Cohort characteristics.

N	50
Age	69.5 ± 6.0
Sex (male)	48 (96%)
**Race**	
White	46 (92%)
Black	3 (6%)
American Indian	1 (2%)
**Smoking status**	
Former	39 (78%)
Current	8 (16%)
Never	3 (6%)
Pack-years	46.4 ± 33.0
FEV_1_% predicted^a^	69.3 ± 20.1
Body mass index	30.5 ± 6.5
**Measurement time point**	
Enrollment	32 (64%)
12-month follow-up	18 (36%)
**Bioimpedance (BIA)**	
BIA-assessed total body percent fat	32.2 ± 6.2
BIA- derived fat mass (kg)^b^	31.1 ± 12.9
BIA-derived fat-free mass (kg)^b^	61.9 ± 10.4
**Dual X-ray absorptiometry (DXA)**	
Total body percent fat	36.7 ± 6.3
Arm % fat	31.7 ± 6.6
Trunk % fat	42.3 ± 7.6
Android % fat	44.8 ± 8.3
Gynoid % fat	37 ± 7.1
Fat mass (kg)	33.6 ± 13.4
Fat-free mass (kg)	55.1 ± 8.2
**Anthropomorphic fat-free mass**^b^	62.6 ± 10.8

Data are expressed as n (%) or mean ± standard deviation.

*BIA* bioimpedance, *DXA* dual X-ray absorptiometry, *FEV*_*1*_ forced expiratory volume in the first second.

^a^n = 48, ^b^n = 47 due to missing anthropomorphic measurements.

**Figure 2:**
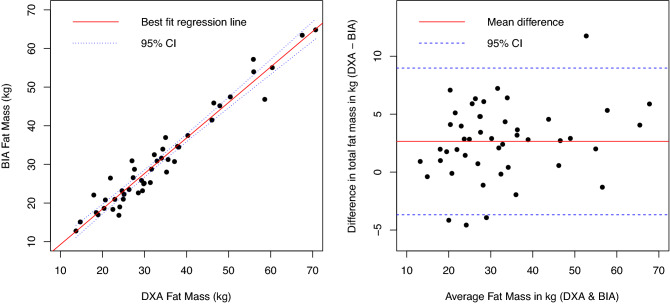
Comparison between bioimpedance (BIA)-derived and dual X-ray absorptiometry (DXA)-assessed fat mass (in kilograms, kg). The left panel is a scatter plot of BIA-derived and DXA-assessed fat mass is shown with the best fit regression (solid red line) and 95% confidence intervals (blue dashed); Spearman ρ = 0.94 (p < 0.0001). The Bland Altman plot is shown in the right panel; average difference between BIA-derived and DXA-assessed fat mass was 2.7 ± 3.2 kg (solid red line); 95% confidence intervals are shown as blue dashed lines.

**Figure 3:**
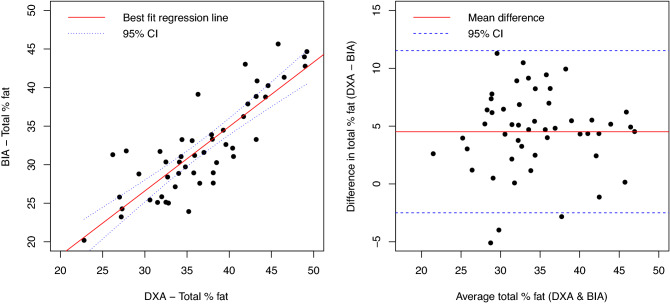
Comparison between bioimpedance (BIA)- and dual X-ray absorptiometry (DXA)-assessed total percent fat. The left panel is a scatter plot of BIA- and DXA-assessed total percent body fat with the best fit regression (solid red line) and 95% confidence intervals (blue dashed); Spearman ρ = 0.80 (p < 0.0001). The Bland Altman plot is shown in the right panel; average difference between BIA-derived and DXA-assessed total percent body fat was 4.5 ± 3.5% (solid red line); 95% confidence intervals are shown as blue dashed lines.

Spearman correlations and the Lin CCC relative to DXA for body composition measurements are shown in Table 2. The correlation between BIA-derived and DXA-assessed fat mass (in kg) was very strong (Spearman's r = 0.94, p < 0.000); the Lin CCC (ρ_c_) also demonstrated excellent concordance (ρ_c_ = 0.95, [95%CI = 0.93, 0.98])—Fig. 2, left panel. As shown in the Bland Altman plot, BIA underestimated the fat mass when compared to the DXA scan, with a mean difference of 2.7 ± 3.2 kg (Fig. 2, right panel). In contrast, while the correlation between BIA- and DXA-assessed total percent body fat was strong (ρ = 0.80, p < 0.0001, Fig. 3, left panel), the Lin CCC (ρ_c_) demonstrated only moderate concordance (ρ_c_ = 0.67, [95%CI = 0.55, 0.79]. BIA underestimated the percent total body fat when compared to the DXA scan, with a mean difference of 4.5 ± 3.5% (Fig. 3, right panel). BIA-assessed and anthropomorphic FFM were strongly correlated with DXA-assessed FFM (Table 2) and were strongly correlated with each other (Spearman’s r = 0.96), but demonstrated only moderate concordance with DXA-assessed FFM using the Lin CCC.

**Table 2:** Spearman correlations and Lin’s concordance correlation coefficients (CCC) between bioimpedance (BIA) and anthropomorphic body composition measurements relative to dual X-ray absorptiometry (DXA).

	Spearman’s ρ	Lin’s CCC [95% CI]
BIA-Percent total body fat	0.80	0.67 [0.55–0.79]
BIA-Fat mass (kg)	0.94	0.95 [0.92–0.98]
BIA-Fat-free mass (kg)	0.91	0.74 [0.65–0.83]
Anthropomorphic fat-free mass (kg)	0.91	0.7 [0.59–0.8]

All p-values were < .0001.

### Correlations by regional percent body fat

Correlations between BIA-assessed total percent fat and DXA-assessed regional percent body fat measurements from the arms, trunk, android, gynoid, and leg regions of the body are shown in Table 3. The strongest correlation between regional percent body fat from the DXA scan and BIA-assessed total percent body fat was observed for percent arm fat measurements. Percent android fat measurements obtained from the DXA scan were the least correlated with the percent total body fat measurements obtained from the BIA device.

**Table 3:** Spearman correlations between BIA-assessed total percent body fat and DXA-assessed regional percent body fat.

Regional Percent Fat	Spearman’s ρ
Arm percent fat	0.827
Trunk percent fat	0.698
Android percent fat	0.621
Gynoid percent fat	0.810
Leg percent fat	0.771

*BIA* bioimpedance, *DXA*  dual X-ray absorptiometry.

All p-values for correlation were < .0001.

### Associations by obesity status

The correlation between BMI and BIA-assessed percent total body fat measurements (Spearman's rho = 0.843; p < 0.001) was comparable to the correlation between BMI and DXA-assessed percent total body fat measurements (Spearman’s rho = 0.802; p < 0.001). Correlations between BIA- and DXA-assessed fat-free mass, fat mass, total percent fat, and regional percent fat measurements, stratified by obesity status, are shown in Table 4. Significantly stronger correlations were observed amongst obese subjects relative to non-obese subjects for both fat mass and total percent fat as well as several regional percent fat measurements. A test for formal interaction between obesity status and total percent fat approached significance (p_interaction_ = 0.054); the interaction between obesity status and fat mass was not significant (p_interaction_ = 0.23).

**Table 4:** Spearman correlations between dual X-ray absorptiometry (DXA) and bioimpedance (BIA)-assessed body composition by obesity status.

	Not obese	Obese
N	31	19
BMI ± SD	26.4 ± 2.5	37.2 ± 5.1
Fat-free mass (kg)^a^	0.86 (< 0.0001)	0.92 (< 0.0001)
Fat mass (kg)^a^	0.84 (< 0.0001)	0.96 (< 0.0001)*
Total body fat %	0.50 (0.0004)	0.85 (< 0.0001)*
Trunk fat %	0.38 (0.03)	0.76 (0.0002)
Android fat %	0.32 (0.08)	0.60 (0.007)
Gynoid fat %	0.46 (0.01)	0.85 (< 0.0001)*
Arm fat %	0.57 (0.0008)	0.87 (< 0.0001)*
Leg fat %	0.33 (0.07)	0.75 (0.0002)*

Data are shown as Spearman’s rho (p-value) unless otherwise noted. “Obese” was defined as a BMI ≥ 30.

*Denotes correlation is significantly different from “Not Obese” group (p < 0.05).

^a^n_non-obese_ = 29, n_obese_ = 18.

## Discussion

Our results support that body composition measurements from a portable, handheld BIA device (Omron HBF-306C) demonstrated (1) excellent intra- and inter-device reproducibility and (2) a strong positive correlation with the body fat measures obtained from DXA scan. BIA-derived fat mass (in kg) demonstrated the strongest concordance with DXA-assessed fat mass, while total percent fat and fat-free mass measurements performed less well. These data support that an inexpensive, commercially-available BIA device can serve as an accurate alternative to assessing differences body fat mass between COPD patients.

Limited data exist on the use of handheld BIA devices, specifically, to assess body fat differences in COPD patients. Previous studies in COPD populations have utilized different types of BIA devices (such as multi-frequency, 8-point stand-on BIA, eight-lead 12-channel isolated switch BIA) to measure body composition differences. For example, a study performed by Ling et al. found that the Direct Segmental Multifrequency-BIA (DSM-BIA) overestimates percent fat mass relative to the DXA scan in middle-aged participants^12^. In contrast, ​ two​ COPD studies found that a tetrapolar and an eight-contact electrode BIA device underestimated fat free mass (FFM) values when compared to the DXA scan in COPD patients^16,17^; but that, similar to our findings, their BIA devices served as a valid alternative to measuring body composition differences. Within our cohort, the finding that BIA-assessed total percent fat was most strongly correlated with DXA-assessed arm fat percent suggests that electrode placement in BIA contributes to regional differences in fat mass estimation; however, the strong correlation with total body fat suggests that arm fat percentage may be a reasonable representation of overall adiposity in a given individual.

In addition to differences in regional fat estimates by lead placement, subject positioning may influence BIA measurements. A study examining a single-frequency, dual lead BIA system with hand-to-foot lead placement found that when lying and standing bioimpedance were compared, there was a measurable and predictable change in body resistance in subjects regardless of age, sex and body size^18^. Furthermore, changes in bioimpedance were dependent upon the time spent in each position; these observations were attributed the changes in resistance to shifts in hydrostatic fluid^18^. The degree to which positional changes impact hand-to-hand BIA systems, such as the one used in our study, is not known and will be investigated in future studies.

Both BIA- and DXA-assessed percent total body fat measures were strongly correlated with BMI, a finding suggestive that simultaneous changes in adiposity and muscle mass may occur in COPD patients. This finding is supported by the lack of a skewed distribution on our Bland–Altman plots. Notably, our device demonstrated *better* performance in estimating fat mass and percent fat among obese individuals relative to non-obese individuals. This is in contrast to previous studies which suggest BIA performs less well at the extremes of BMI^6^. We hypothesize the difference in performance among obese participants in our study may be attributable to the device used in our study.

We acknowledge several limitations to this study. First, the predominance of males of European ancestry may limit the generalizability of our results to populations which include females and individuals of different ancestries and ethnicities. Additionally, participants in our study were enrolled in a physical activity intervention and may not accurately represent those of the general COPD population (self-selection bias). Second, although subjects with implanted electronic devices were excluded from our study, individuals with a history of non-electronic metal implants (e.g., joint replacements) were *not* excluded. A post-hoc review of the medical records identified 7 subjects with metallic orthopedic implants (6 lower extremity, 1 laminectomy, none in the upper extremities); no significant differences in DXA- or BIA-assessed total percent body fat by orthopedic implant status were found. Third, because our subjects were derived from clinical trial of exercise and physical activity targeting an older and potentially frail population, subjects were not required to fast prior to DXA and BIA measurements. Retrospective analysis of data available from 41 subjects on the time since their last meal demonstrated a median fasting period of 6–8 h, with 2 subjects (< 5%) reporting a meal within 2 h of assessment. We acknowledge the potential for additional variability introduced by the lack of systematic fasting prior to body composition assessment, however, because our primary comparison was between DXA and BIA (both of which were taken in the same fasting/non-fasting state), we assert that our results remain valid. Fourth, because the handheld BIA device demonstrated only moderate concordance with DXA for FFM, a measure which has been shown to correlate with clinical outcomes in COPD^19,20^, the utility of our BIA-derived FFM and outcomes in our population may be limited. Fifth, we acknowledge that, due to small sample size, our power to detect associations and interactions may be limited. However, we wish to highlight that, *despite* the modest sample size, we had sufficient power to detect robust and highly statistically significant associations between BIA and DXA which support our conclusion that a handheld BIA device may offer insight into body composition differences between COPD patients. Future studies are needed to assess the reproducibility and generalizability of our findings.

## Data Availability

Due to institutional policies governing the data examined in this work, requests for data will be reviewed and must be approved by the local IRB prior to release. Investigators should contact the corresponding author for guidance and assistance with the data request process.

## Abbreviations

BIA: Bioimpedance
BMI: Body mass index
COPD: Chronic obstructive pulmonary disease
DXA: Dual X-ray absorptiometry
FFM: Fat-free mass
